# Peroxisome Proliferator-Activated Receptor-γ (PPAR-γ) Agonists Attenuate the Profibrotic Response Induced by TGF-β1 in Renal Interstitial Fibroblasts

**DOI:** 10.1155/2007/62641

**Published:** 2007-12-17

**Authors:** Weiming Wang, Feng Liu, Nan Chen

**Affiliations:** Department of Nephrology, Ruijin Hospital, Shanghai Jiao Tong University School of Medicine, Shanghai 200025, China

## Abstract

*Background.* Studies have shown that peroxisome proliferator-activated receptor-γ (PPAR-γ) agonists could ameliorate renal fibrotic lesions in both diabetic nephropathy and nondiabetic chronic kidney diseases. In order to elucidate the antifibrotic mechanism of PPAR-γ agonists, we investigated the effects of PPAR-γ activation on TGF-β1-induced renal interstitial fibroblasts. *Methods.* In rat renal interstitial fibroblasts (NRK/49F), the mRNA expression of TGF-β1-induced α-smooth muscle actin (α-SMA), connective tissue growth factor (CTGF), fibronectin (FN) and collagen type III (Col III) were observed by reverse transcriptase-polymerase chain reaction (RT-PCR). The protein expressions of FN and Smads were observed by Western blot. *Results.* In NRK/49F, TGF-β1 enhanced CTGF, FN and Col III mRNA expression in a dose- and time-dependent manner. α-SMA, CTGF, FN and Col III mRNA and FN protein expression in 15-deoxy-$\Delta^{12,14}$-prostaglandin J2 (15d-PGJ2)-troglitazone- and ciglitazone-pretreated groups, respectively, were significantly decreased compared with the TGF-β1-stimulated group. TGF-β1 (5 ng/mL) enhanced p-Smad2/3 protein expression in a time-dependent manner. Compared with the TGF-β1-stimulated group, p-Smad2/3 protein induced by TGF-β1 in PPAR-γ agonists-pretreated groups significantly decreased with no statistical difference amongst the three pretreated groups. *Conclusion.* PPAR-γ agonists could inhibit TGF-β1-induced renal fibroblast activation, CTGF expression and ECM synthesis through abrogating the TGF-β1/Smads signaling pathway.

## 1. INTRODUCTION

Tubulointerstitial fibrosis (TIF) is the final manifestation of end stage renal disease (ESRD) [1] and renal injury is correlated to the degree of renal interstitial fibrosis. One of the major pathological characteristics of TIF is the activated tubulointerstitial fibroblasts transdifferentiating into myofibroblasts. Furthermore, extracellular matrix (ECM) secreted by fibroblasts deposits in the tubular interstitium and results in interstitial fibrosis. Transforming growth factor-*β* (TGF-*β*) is known to be one of the major mediators that lead to fibrosis. Connective tissue growth factor (CTGF) has been receiving more and more attention for being one of the major downstream mediators of TGF-*β*. Peroxisome proliferator-activated receptor *γ* (PPAR-*γ*) is a member of the ligand-activated nuclear transcriptional superfamily and is expressed in several tissues which includes kidney [2–5]. PPAR-*γ* is activated by its ligand, 15-deoxy-Δ^12,14^-prostaglandin J_2_ (15d-PGJ_2_), or activators (synthetic thiazolidinedione PPAR-*γ* agonists) and then participates in the regulation of cellular function [2]. Apart from maintaining glucose homeostasis, it also plays an important role in inflammation and cell cycles. Studies have shown that PPAR-*γ* could control glomerular inflammation, modulate vasodilator substances like prostaglandins and NO [6, 7], antagonize glomerulosclerosis of diabetic nephropathy, and improve renal function. In a rat remnant kidney model of renal fibrosis, administration of the PPAR-*γ* agonist troglitazone is associated with reduction of proteinuria, improved serum creatinine, and glomerulosclerosis [8]. PPAR-*γ* agonists could exert antifibrotic effects on human PTC in high glucose levels by attenuating the production of AP-1, TGF-*β*1, and the downstream production of the extracellular matrix protein fibronectin [8, 9]. PPAR-*γ* activation also decreases glomerular cell proliferation and suppresses plasminogen activator inhibitor-1 (PAI-1) and TGF-*β* expression [10]. Furthermore, it down-regulated TGF-*β*1-induced fibronectin expression in mouse glomerular mesangial cells by inhibiting activator protein-1 (AP-1) [11–13]; but the relationship between PPAR-*γ* and tubulointerstitial fibrosis is not clear yet. By studying the effects of PPAR-*γ* activation on TGF-*β*-induced fibrosis and its mechanism, our study demonstrates the potential perspective for the antifibrotic property of PPAR-*γ* agonists.

## 2. MATERIALS AND METHODS

**Cell culture and treatments**

NRK-49F, the immortalized rat kidney interstitial fibroblast cells were obtained from the Chinese Academy of Sciences. Cells were maintained in DMEM/F-12 medium supplemented with 10% heat-inactivated fetal calf serum (Gibco/BRL) in a humidified atmosphere of 5% CO_2_/95% air at 37°C. The cells were passaged every 4 days and then harvested onto six-well culture plates to 60–70% confluence in the complete medium for 16 hours followed by changing to serum-free medium. TGF-*β*1, 15d-PGJ2, troglitazone, and ciglitazone (BIOMOL Research Laboratories, Inc., Plymouth Meeting, Pa) were freshly dissolved in culture media and added to the cultures at the indicated concentrations and for the indicated time periods.

**RNA isolation and Reverse transcriptase-polymerase chain reaction (RT-PCR)**

Total RNA from NRK/49F cells was isolated by using a TRIzol extraction kit (Gibco/BRL) according to the manufacturer’s directions. First-strand cDNA was synthesized using 2 *μ*g of RNA in 20 *μ*L of reaction buffer by reverse transcription using Moloney murine leukemia virus-derived reverse transcriptase (Promega). Complementary DNA was amplified in 100 *μ*L total volume which contained 50 mmol/L KCl, 20 mmol/L Tris-HCl (pH 8.0), 10 mmol/L deoxynucleoside triphosphate (dNTP), 1.5 mmol/L MgCl2, 1U *Taq* polymerase, and 10 pmol of specific PCR primers. Table 1 showed the sets of primers used for PCR amplification. Beta-actin was amplified and yielded a 202 bp PCR product as the internal standard. The number of cycles used allowed quantification without saturation. Amplification products were separated by electrophoresis on 1.2% agarose gel, followed by ethidium bromide staining, and then photographed. The amplification bands were quantitated from negative by scanning densitometry. Semiquantitation was done by serial dilution of the input cDNA to measure the mRNA. The proportion of specific gene product to *β*-actin product was used for semiquantitive analysis.

**Western blotting**

Extracted protein was loaded on a 10% SDS-PAGE gel and transferred onto nitrocellulose membranes. Then proteins were then blocked and incubated with specific antibodies: *β*-actin (Sigma-Aldrich), fibronectin (GIBco), type III collagen (Sigma), Smad2/3, or p-smad2/3 (Santa Cruz Biotechnology, Calif, USA). Membranes were subsequently washed, incubated with specific secondary horseradish peroxidase—conjugated antibodies, and revealed with the enhanced chemiluminescence (ECL) kit (Life Science Products, Boston, Mass, USA). The band intensity was analyzed by scanning densitometry.

**Statistical analysis**

All experiments were repeated at least three times, and the results are presented as mean ± standard deviation (SD). All data were analyzed by SAS 6.12. ANOVA and t-test were performed for statistical analysis as appropriate. A *P* value less than .05 was considered to be statistically significant.

## 3. RESULTS

TGF-*β*1-induced CTGF, FN, and Col III mRNA expression in a dose-dependent and time-dependent manner in NRK/49F cells.

**Figure 1:**
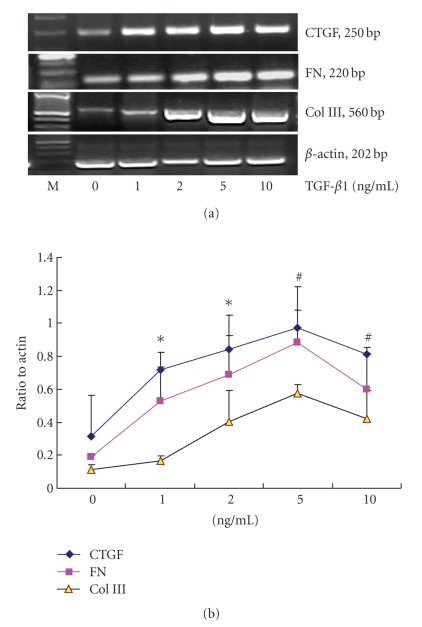
TGF-*β*1-induced CTGF, FN, Col III mRNA expression in a DOSE-dependent manner, *P < .05 versus control; #*P* < .01 versus control (n=3 for each group).

**Figure 2:**
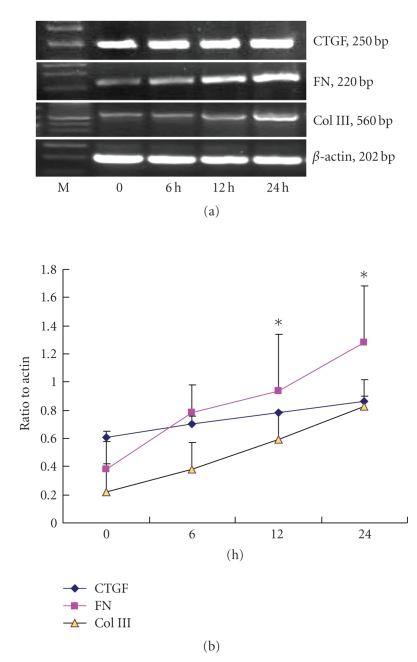
TGF-*β*1-induced CTGF, FN, Col III mRNA expression in a time-dependent manner, *P < .05 versus control; effects of TGF-*β*1 on CTGF, FN, Col III mRNA expression in NRK/49F. NRK/49Fs were cultured in the 25 cm^2^ culture flash by 1 × 10^6^ which contains DMEM/F12 medium with 10% fetal bovine serum, and cultured in the 37°C, 5%CO_2_ incubator. When the cells were 80–90% confluent, continued to culture in the DMEM/F12 medium without serum for 24 hours to synchronize cell growth. Different dosages of TGF-*β*1 (0, 1, 2, 5, 10 ng/mL) were added to the medium and the cells were cultured for another 24 hours, or TGF-*β*1 was added (5 ng/mL) to the medium and cultured for different times (0, 6, 12 and 24 hours). Both control and experimental groups repeated for 3 times.

The induction of CTGF and ECM expression is a hallmark of renal fibrosis in many types of primary glomerular disease. Therefore, we first examined the effect of TGF-*β*1 on CTGF and ECM expression in cultured NRK/49F cells. As shown in Figures 1and 2, NRK/49F cells had basal expression of CTGF, FN, and Col III mRNA. After stimulating with different concentration of TGF-*β*1, the expression of CTGF, FN, and Col III mRNA increased significantly in a dose-dependent manner (see Figure 1). After stimulating at 1 ng/mL TGF-*β*1 for 1 hour, the expression of CTGF, FN, and Col III mRNA began to increase (*P* < .05), peaked at 5 ng/mL (*P* < .01), and decreased at 10 ng/mL but was still greater than that of the control group (*P* < .01). As shown in Figure 2, TGF-*β*1 also induced CTGF, FN, Col III mRNA expression in a time-dependent manner in NRK/49F cells. After stimulating for 6 hours with TGF-*β*1 (5 ng/mL), expression of CTGF, FN, and Col III mRNA increased, and peaked at 24 hours (*P* < .01).

**Effects of PPAR-*γ* agonists on *α*-SMA, CTGF, Col III, and FN mRNA expression**

**Figure 3:**
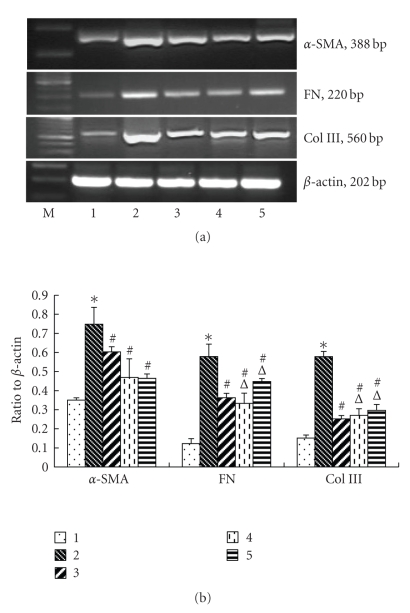
Effects of PPAR-*γ* agonists on TGF-*β*1-induced *α*-SMA, FN, Col III mRNA expression. Cells were cultured as described above and pretreated by 10 uM 15d-PGJ2, troglitazone, and ciglitazone for 2 hours and then added 5 ng/mL TGF-*β*1. Set a blank control group and 5 ng/mL TGF-*β*1 stimulating group, cultured for 24 hours and then harvested the cells. Both control and experimental groups repeated for 3 times. (1) control, (2) 5 ng/mL TGF-*β*1 group, (3) 15d-PGJ2 + 5 ng/mL TGF-*β*1 group, (4) troglitazone + 5 ng/mL TGF-*β*1 group, (5) ciglitazone + 5 ng/mL TGF-*β*1 group, *P < .05 versus control, #*P* < .05 versus 5 ng/mL TGF-*β*1 group, △ *P* < .05 versus 15d-PGJ2 group.

**Figure 4:**
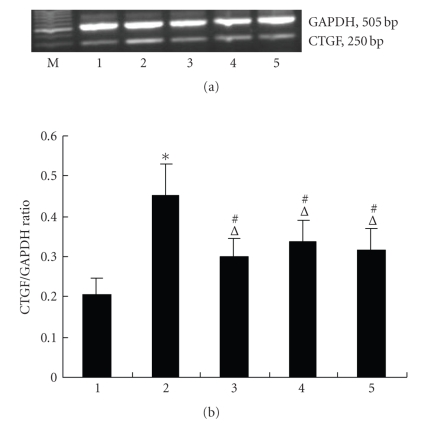
Effects of PPAR-*γ* agonists on TGF-*β*1-induced CTGF mRNA expression (*n* = 3 for each group), (1) control, (2) 5 ng/mL TGF-*β*1 group, (3) 15d-PGJ 2+5 ng/mlTGF-*β*1 group, (4) troglitazone + 5 ng/mL TGF-*β*1 group, and (5) ciglitazone + 5 ng/mL TGF-*β*1 group, *P* < .05 versus control, #*P* < .05 versus 5 ng/mL TGF-*β*1 group, △ *P* < .05 versus 15d-PGJ2 group.

The induction of *α*-SMA is a hallmark of renal interstitial fibroblast activation. Therefore, we examined the effect of PPAR-*γ* activation on *α*-SMA, CTGF, Col III, and FN mRNA expression in cultured NRK/49F. As shown in Figures 3 and 4, 10 μM 15d-PGJ2 dramatically suppressed TGF-*β*1-mediated *α*-SMA, CTGF, Col III, and FN mRNA expression (*P* < .05). Similarly, the synthetic PPAR-*γ* agonists troglitazone, and ciglitazone also effectively inhibited TGF-*β*1-mediated *α*-SMA, CTGF, Col III, and FN mRNA expression in NRK/49F. Such expression in the troglitazone- and ciglitazone-treated groups was less than that in the 15d-PGJ2 treated group.

**Effects of PPAR-*γ* agonists on TGF-*β*-induced FN protein expression**

**Figure 5:**
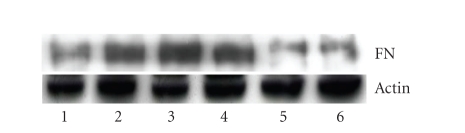
Effects of PPAR-*γ* on TGF-*β*1-induced FN protein expression. Cells were cultured in the methods described above. Set groups: (1) control group (without TGF-*β*1), (2) added 2 ng/mL TGF-*β*1; (3) 5 ng/mL TGF-*β*1 group; (4) 10 uM 15d-PGJ2 + 5 ng/mL TGF-*β*1 group; (5) 10 uM Troglitazone + 5 ng/mL TGF-*β*1 group; (6) 10 uM ciglitazone + 5 ng/mL TGF-*β*1 group (all the three reagents were pretreated for 2 hours). All the cells in each group were cultured for 24 hours and then harvested cells, extracted total protein (results of one of the three repeated experiments).

Figure 5 demonstrates that 15d-PGJ2, troglitazone, and ciglitazone suppressed TGF-*β*1-mediated fibronectin expression in NRK/49F cells. NRK/49F cells expressed a considerable amount of fibronectin at basal conditions, and TGF-*β*1 significantly induced FN expression. However, NRK/49F treated with 15d-PGJ2, troglitazone, and ciglitazone counteracted the TGF-*β*1-stimulated overproduction of fibronectin. However, the level of fibronectin in five PPAR-*γ* agonists treated groups was not significantly different (*P* > .05).

**Effects of TGF-*β*1 on Smads protein expression in NRK/49F**

**Figure 6:**
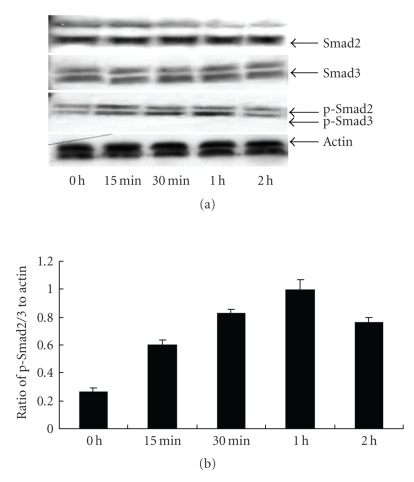
Effects of TGF-*β*1 on Smad2/3, p-smad2/3 protein expression in NRK/49F (results of one of three repeated experiments).

Figure 6 shows that NRK/49F had basal expression of phosphorylated smad2/3 protein. After stimulating by TGF-*β*1 for 15 minutes, p-Smad2/3 protein expression began to increase, peaked at 1 hour, then began to decrease at 2 hours. There was no difference of total Smad2/3 protein expression.

**Effects of PPAR-*γ* on TGF-*β*/Smads protein expression**

**Figure 7:**
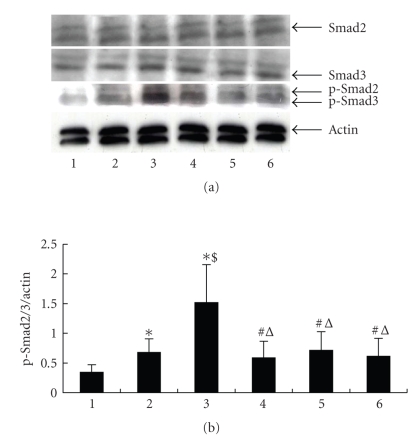
Effects of PPAR-*γ* on TGF-*β*1-induced Smads protein expression (results of one of the three repeated experiments): (1) control, (2) 2 ng/mL TGF-*β*1 group, (3) 5 ng/mL TGF-*β*1 group, (4) 15d-PGJ2 + 5 ng/mL TGF-*β*1 group, (5) troglitazone + 5 ng/mL TGF-*β*1 group, (6) ciglitazone + 5 ng/mL TGF-*β*1 group,

To explore the molecular mechanism by which PPAR-*γ* agonists inhibit TGF-*β*1-mediated renal fibroblast activation, we studied the effect of 15d-PGJ2, troglitazone, and ciglitazone on Smad signalling pathway. As shown in Figure 7, pretreatment of NRK/49F fibroblasts with 15d-PGJ2, troglitazone, and ciglitazone was able to block Smad phosphorylation. There was no difference in each interfering group (*P* > .05). Total Smad2/3 protein of each group showed no difference.

## 4. DISSCUSSION

Tubulointerstitial fibrosis (TIF) is the final manifestation of end stage renal disease (ESRD). The major pathological changes of TIF are the proliferation of interstitial fibroblasts, transdifferentiation of fibroblast into myofibroblasts (the major characteristic of which is the increase of *α*-smooth muscular actin expression), and overdepositing of extracellular matrix (ECM) such as fibronectin and collagen type I, type II, and type IV. Of all the cytokines and growth factors involved, TGF-*β*1 plays the most important role. The elevation of TGF-*β*1 expression is closely correlated with glomerulosclerosis and interstitial fibrosis. Anti-TGF-*β*1 antibody and anti-TGF-*β* receptor antibody could reduce the production of ECM [14]. As TGF-*β*1 has many biological effects, suppression of TGF-*β*1 expression/activation or blocking TGF-*β*1 at its receptors could result in many biological side effects. Therefore, targeting the downstream mediators of the over-activated signaling pathway is a good way to antagonize the progression of ESRD and thus become the prime focus of current studies.

Connective tissue growth factor (CTGF) is one of the downstream mediators of TGF-*β*1-induced fibrosis and it participates in fibroblast proliferation and adhesion and in inducing ECM production. Myofibroblasts transdifferentiated from renal interstitial fibroblasts are major sources of tubulointerstitial ECM. Our study shows that NRK/49F had basal expression of α-SMA mRNA until it is stimulated by TGF-*β*1, whereupon its expression increased significantly, demonstrating that TGF-*β*1 could induce tubulointerstitial fibroblasts to transdifferentiate into myofibroblasts. TGF-*β*1 could increase the mRNA expression of the downstream mediator CTGF and major constituents of ECM (collagen type III and fibronectin) in a time-dependent and dose-dependent manner. Our study demonstrates that TGF-*β* is one of the major fibrosis-inducing mediators which could induce transdifferentiation of renal fibroblasts and production of ECM. Recently, study by Lin et al. showed that activation of Smad3/4 was essential for TGF-*β*1–induced CTGF transcription and that pentoxifylline (PTX), a potent inhibitor of CTGF, could inhibit CTGF expression by interfering with Smad3/4-dependent CTGF transcription through protein kinase A and block the profibrogenic effects of CTGF on renal cells [28].

PPAR-*γ* has now become the therapeutic target of research on kidney diseases like diabetic nephropathy, glomerulosclerosis, glomerulonephritis, and hypertensive nephropathy [2]. 15d-PGJ2 is the natural ligand of PPAR-*γ*, and thiazolidinediones (TZDs) such as troglitazone, and rosiglitazone are its agonists. It has been proven in animal models (streptozotocin-induced diabetic nephropathy models and 5/6 nephrectomized models) that TZDs could reduce the expression of extraglomerular matrix and ameliorate renal injury [2]. TZDs and 15d-PGJ_2_ could reduce production of collagen type I and fibronectin in rats [15, 16], and inhibit production of proinflammatory cytokines and chemotactic factors (e.g., NO, COX-2, MCP-1, etc.) [17, 18]. Tubulointerstitial fibroblasts could express basal amounts of PPAR-*γ*. Our unpublished study showed that PPAR-*γ* could inhibit proliferation of human tubulointerstitial fibroblasts and trigger their apoptosis. However, there are few studies on PPAR-*γ* agonists and production of ECM. Our study found that 15d-PGJ2, troglitazone, and rosiglitazone could reduce TGF-*β*-induced production of *α*-SMA and ECM (collagen type III and fibronectin) and inhibit expression of CTGF mRNA. These results show that PPAR-*γ* agonists have an antifibrotic effect through inhibition of TGF-*β*-induced renal fibroblast transdifferentiation and ECM production. Such results are similar to those results of studies on glomerulosclerosis and arteriosclerosis [19–21]. Exposure of human cortical fibroblasts to pioglitazone causes an antiproliferative effect and reduces ECM production through mechanisms that include reducing TIMP activity independent of TGF-*β*1 [22].

Different PPAR-*γ* ligands or agonists may have different mechanisms in different cells and there are also PPAR-*γ* independent pathways involved. Baoling found that pioglitazone, and 15d-PGJ2 could inhibit expression of fibronectin induced by TGF-*β* in rats and the effects of pioglitazone are due to activation of PPAR-*γ*. However, there were PPAR-*γ*-independent pathways involved in the mechanism of 15d-PGJ2 action [16]. Whether PPAR-*γ* inhibits TGF-*β*1-induced fibrosis by activating intracellular PPAR-*γ*-receptors requires further study. Different PPAR-*γ* agonists may even have opposite effects. Sawano’s study shows that 15d-PGJ2 could downregulate IL-1*β*-induced COX-2 expression and troglitazone/rosiglitazone could not reduce the expression of COX-2 [17]. Panzer found that in experimental glomerulonephritis induced by ATS (antithymus antibody), troglitazone, and ciglitazone could upregulate MCP-1 expression and increase monocyte/macrophage infiltration and adhesion, however, 15d-PGJ2 has no effect on MCP-1 expression [23]. Our study shows that troglitazone, and ciglitazone have a more inhibitory effect on *α*-SMA expression than 15d-PGJ2. Comparing the intervention of different reagents, the expression of CTGF, collagen type III, and fibronectin shows no difference. Such results suggest that these reagents have similar effects on fibrosis.

Phosphorylated Smad2/Smad3 (p-Smad2/3) is the main signaling pathway of TGF-*β*1 and it participates in the biological effects of TGF-*β* which include cell proliferation, inflammation reaction, and fibrosis [24, 25]. They are essential mediators of TGF-*β*-induced endothelial cell transdifferentiation and ECM and CTGF expression [26]. Studies have shown that the TGF-*β*/Smads signaling pathway participates in many pathophysiological processes related to kidney diseases like diabetic nephropathy, glomerulonephritis and glomerulosclerosis [27]. Our study shows that TGF-*β*1 induces Smad2/3 phosphorylation in a time-dependent manner, which suggests that TGF-*β* could induce Smad2/3 phosphorylation in renal fibroblasts. 15d-PGJ2, troglitazone, and ciglitazone could reduce TGF-*β*-induced p-Smad2/3 protein expression while the total amount of Smad2/3 protein did not change. Such results suggest PPAR-*γ* agonists could inhibit the fibrotic effect of TGF-*β* by interfering in the phosphorylation of Smad2/3. Moreover, all three reagents show no significant difference in inhibiting phosphorylation of Smad2/3. Our study suggests that Smad 2/3 signaling pathway is essential in antifibrotic effects of PPAR-*γ* agonists. While in the study by Yang et al., Smad 2/3 phosphorylation was not inhibited by hepatocyte growth factor (HGF), which acts as a potent inhibitor of the TGF-*β*1-mediated myofibroblastic activation [29] Therefore, further study is required to investigate role of Smad signaling pathway in different inhibitors of TGF-*β*1-mediated myofibroblastic activation.

In conclusion, PPAR-*γ* could antagonize TGF-*β*-induced fibrosis by interfering TGF-*β*/Smad signaling pathway. Such results suggest a perspective for the antifibrotic effects of PPAR-*γ* and such effects may become the therapeutic target of ESRD.

## Figures and Tables

**Table 1:** PCR primer sequence and conditions.

Name		Primer sequence	Fragment length
*α*-SMA	Upstream	5′-GATCACCATCGGGAATGAACGC-3′	388 bp
	Downstream	5′-CTTAGAAGCATTTGCGGTGGAC-3′	
CTGF	Upstream	5′-GAGCTTTCTGGCTGCACC-3′	250 bp
	Downstream	5′-TCTCCGTACATCTTCCTG-3′	
FN	Upstream	5′-TTATGACGATGGGAAGACCTA-3′	220 bp
	Downstream	5′-GTGGGGCTGGAAAGATTACTC-3′	
Col III	Upstream	5′-CTGGACCAAAAGGTGATGCTG-3′	560 bp
	Downstream	5′-TGCCAGGGAATCCTCGATGTC-3′	
GAPDH	Upstream	5′-GACAAGATGGTGAAGGTCGG-3′	505 bp
	Downstream	5′- CATGGACTGTGGTCATGAGC-3′	
*β*-actin	Upstream	5′- TGGAGAAGAGCTATGAGCTGCCTG-3′	202 bp
	Downstream	5′- GTGCCACCAGACAGCACTGTGTTG-3′	
